# Meta‐analysis: Interleukin 6 gene ‐174G/C polymorphism associated with type 2 diabetes mellitus and interleukin 6 changes

**DOI:** 10.1111/jcmm.16575

**Published:** 2021-05-07

**Authors:** Hao Cheng, Wenbin Zhu, Mou Zhu, Yan Sun, Xiaojie Sun, Di Jia, Chao Yang, Haitao Yu, Chunjing Zhang

**Affiliations:** ^1^ Department of Clinics Qiqihar Medical University Qiqihar China; ^2^ Department of Molecular Biology Laboratory Qiqihar Medical University Qiqihar China; ^3^ Department of Biochemistry and Molecular Biology Qiqihar Medical University Qiqihar China; ^4^ Department of Clinical Pathogen Microbiology Qiqihar Medical University Qiqihar China; ^5^ Department of Clinical Biochemistry Qiqihar Medical University Qiqihar China; ^6^ Department of Cell Biology Qiqihar Medical University Qiqihar China

**Keywords:** interleukin 6, polymorphism, risk, type 2 diabetes mellitus

## Abstract

The gene coding interleukin 6 (*IL‐6*) is a promising candidate in predisposition to type 2 diabetes mellitus (T2DM). This study aimed to meta‐analytically examine the association of *IL‐6* gene −174G/C polymorphism with T2DM and circulating IL‐6 changes across −174G/C genotypes. Odds ratio (OR) and standard mean difference (SMD) with 95% confidence interval (CI) were calculated. Twenty‐five articles were meta‐analysed, with 20 articles for T2DM risk and 9 articles for circulating IL‐6 changes. Overall, there was no detectable significance for the association between −174G/C polymorphism and T2DM, and this association was relatively obvious under dominant model (OR: 0.82, 95% CI: 0.56‐1.21). Improved heterogeneity was seen in some subgroups, with statistical significance found in studies involving subjects of mixed races (OR: 0.63, 95% CI: 0.46‐0.86). Begg's and filled funnel plots, along with Egger's tests revealed week evidence of publication bias. In genotype‐phenotype analyses, carriers of −174CC and −174CG genotypes separately had 0.10 and 0.03 lower concentrations (pg/mL) of circulating IL‐6 than −174GG carriers. Albeit no detectable significance for the association of −174G/C with T2DM, our findings provided suggestive evidence on a dose‐dependent relation between −174G/C mutant alleles and circulating IL‐6 concentrations, indicating possible implication of this polymorphism in the pathogenesis of T2DM.

## 1 INTRODUCTION

Diabetes is a chronic metabolic disorder, and globally an estimated 422 million persons are affected by diabetes, mainly in low‐ and middle‐income countries.^1^ The most common is type 2 diabetes mellitus (T2DM), which accounts for 90% to 95% of all diabetes. T2DM is a complex, multifactorial disease, attributing to the interaction between genetic defects and environmental factors.^2^, ^3^ As a risk factor of nearly all‐cause mortality, T2DM can affect people across different life stages.^4^ So, early identification of persons at a higher risk for T2DM is of great clinical and public health importance.

It is well known that T2DM is a polygenic disease. Extensive efforts have been made to decipher the genetic basis of T2DM, especially with the advent of genome‐wide association studies (GWASs).^5^, ^6^, ^7^ Although over a hundred genetic variants in predisposition to T2DM have been characterized, only a modest portion of T2DM heritability can be interpreted.^8^, ^9^ One of the major challenges facing global geneticists is the inconsistent replication of candidate genes with biological implications across different populations.^10^, ^11^ The gene coding interleukin 6 (*IL‐6*) is one such gene.

Biologically speaking, IL‐6 can induce the development of insulin resistance and pathogenesis of T2DM via regulating inflammatory responses.^12^, ^13^ A promoter polymorphism in *IL‐6* gene, −174G/C or rs1800795, has been extensively studied in association with T2DM, yet the results of most prior studies are poorly replicated.^14^, ^15^, ^16^, ^17^, ^18^, ^19^, ^20^, ^21^, ^22^, ^23^, ^24^ The underlying reasons are manifold, likely involving differences in genetic backgrounds, study designs and statistical power, as well as baseline characteristics of diverse populations.

To shed some light upon these reasons and yield more information for future investigations, we here prepared a systematic review of published studies to meta‐analytically examine the association of *IL‐6* gene −174G/C polymorphism with T2DM, as well as the changes of circulating IL‐6 concentrations across −174G/C genotypes. Meanwhile, the possible sources for between‐study heterogeneity attributed to inconsistent observations were also interrogated.

## 2 METHODS

This meta‐analysis was proceeded in accordance with the guidelines of the Preferred Reporting Items for Systematic Reviews and Meta‐analyses (PRISMA) statement.^25^ The PRISMA checklist is presented in Table S1.

### 2.1 Search strategy

Public databases including Medline/PubMed, EMBASE (Excerpta Medica database) and Web of Science were reviewed to seek potentially qualified articles published prior to 8 September 2020. Key terms for literature search were (‘interleukin 6’ OR ‘IL‐6’ OR ‘inflamma*’ OR ‘cytokine*’) [Title and Abstract] AND (‘diabet*’) [Title] AND (‘SNP’ OR ‘polymorphism’ OR ‘varia*’ OR ‘mutation*’) [Title and Abstract]. Only articles written in the English language and conducted among human participants were retrieved.

In addition, the reference lists of major articles or reviews were scanned for potential missing articles. Search process was independently completed by two of us (Hao Cheng and Wenbin Zhu), by using same key terms aforementioned, and any conflicts were adjudicated by a third author (Chunjing Zhang). The results were integrated, and duplicates were removed from the final reference set.

### 2.2 Eligibility criteria

Eligible articles needed to meet the following three criteria: (i) available genotype or allele counts of *IL‐6* gene −174G/C polymorphism in both T2DM patients and controls or available circulating IL‐6 concentrations across the genotypes of −174G/C polymorphism; (ii) clear definition of T2DM according to official guidelines; (iii) the adoption of validated assaying methods to determine three −174G/C genotypes.

If the retrieved publication was a narrative or quantitative review, was an animal study, focused on diabetic complications, did not have valid control groups, lacked necessary genotype information or was published in the languages other than the English, this publication was excluded from this meta‐analysis.

### 2.3 Data extraction

From each qualified article, extracted data included first author's name, year of publication, race or ethnicity, disease status, T2DM diagnosis, control source, study design, matched condition, age, gender and body mass index, as well as, if available, haemoglobin A1c (HbA1c), fasting plasma glucose (FPG), postprandial glucose (PPG), total cholesterol (TC), triglyceride (TG), high‐density lipoprotein cholesterol (HDLC) and low‐density lipoprotein cholesterol (LDLC). Data extraction process was independently finished by two of us (Hao Cheng and Wenbin Zhu), and disagreement was solved by a third author (Chunjing Zhang).

### 2.4 Statistical analyses

All statistical analyses were performed with the use of STATA software Release 14.1 (StataCorp, College Station, TX, USA).

Weighted odds ratio (OR) and its 95% confidence interval (95% CI) were calculated to assess the association between *IL‐6* gene −174G/C polymorphism and T2DM. In addition, changes in circulating IL‐6 and the other laboratory biomarkers across −174G/C genotypes were expressed as standard mean difference (SMD) and 95% CI. Pooled OR and SMD were derived under the random‐effects model. The inconsistency index (*I* ^2^) was adopted to appraise between‐study heterogeneity, which meant that the percentage of observed variability between studies that was due to heterogeneity instead of a chance finding. If the *I^2^* is over 50.0%, statistically significant heterogeneity is recorded. Subsidiary analyses were done to interrogate underlying sources for between‐study heterogeneity.

Cumulative analyses and sensitivity analyses were carried out to appraise the risk of bias. The former measured the impact of the first publication on subsequent publications and the evolution of cumulative estimates over time. The latter removed one publication at a time to appraise the influence of a single publication on pooled estimates.

Both Begg's plots and filled funnel plots were depicted to appraise the probability of publication bias. If the funnel shape was symmetric and the probability of Egger's tests was over 10%, a low probability of publication bias was recorded.

## 3 RESULTS

### 3.1 Retrieved articles

**FIGURE 1:**
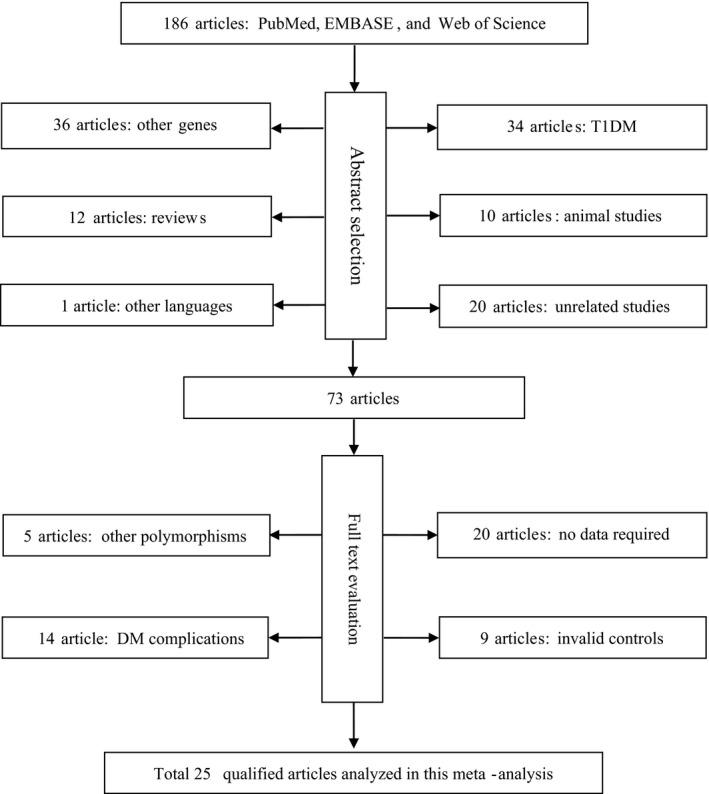
Flow diagram of selection process in the current meta‐analysis

Figure 1 shows the detailed search process for eligible articles. Our initial search of three public databases retrieved a total of 186 articles, and only 25 of them met our pre‐specified inclusion and exclusion criteria. Twenty articles^10^, ^11^, ^40^ including 26 studies with 4,688 patients and 10,700 controls provided data on the association between *IL‐6* gene −174G/C polymorphism and T2DM. Nine articles^10^, ^18^, ^21^, ^26^, ^27^, ^41^, ^42^, ^43^, ^44^ including 12 studies with 4,090 subjects provided data on the changes of circulating IL‐6 concentrations across −174G/C genotypes.

### 3.2 Baseline characteristics of eligible studies

Table 1 provides the baseline characteristics of all eligible studies. All included articles were published during the years between 2003 and 2019. Total sample sizes ranged from 40 to 5840. T2DM was doctors’ diagnosed or according to the ADA (American Diabetes Association) or WHO (World Health Organization) 1999 guidelines.

**TABLE 1:** The baseline characteristics of all involved studies in the current meta‐analysis

First Author	Year	Country	Ethnicity	Disease	Matched	Control status	Diagnosis	Study design	Age (yrs) in cases	Age (yrs) in controls
Fathy, SA (T2DM w/t DKD)	2019	Kuwait	Caucasian	T2DM without DKD	NA	Healthy	Doctor's diagnosis	Retrospective	58.5	53.8
Fathy, SA (T2DM w/o DKD)	2019	Kuwait	Caucasian	T2DM with DKD	NA	Healthy	Doctor's diagnosis	Retrospective	61.0	53.8
Lara‐Gómez, RE	2019	Mexico	Mixed	T2DM	NA	Healthy	Doctor's diagnosis	Cross‐sectional	59.1	36.2
Saxena, M.	2018	India	Indian	T2DM	NA	Healthy	NA	Prospective		
Plataki, MN	2018	Greece	Caucasian	T2DM	NA	Controls (Normal Glucose)	ADA	Retrospective	68.3	74.9
Hameed, I. (T2DM w/t DKD)	2018	India	Indian	T2DM without DKD	NA	Healthy	Doctor's diagnosis	Prospective	54.1	
Hameed, I. (T2DM w/o DKD)	2018	India	Indian	T2DM with DKD	NA	Healthy	Doctor's diagnosis	Prospective	57.9	
Rodrigues, KF	2017	Brasil	Mixed	T2DM	YES	Healthy	ADA	Cross‐sectional	56.0	53.0
Ponnana, M.	2017	India	Indian	T2DM	YES	Healthy	Doctor's diagnosis	Prospective	33.3	30.2
Neelofar, K. (T2DM w/t DKD)	2017	India	Indian	T2DM without DKD	YES	Hospital, Healthy	ADA	Prospective	52.8	50.1
Neelofar, K. (T2DM w/o DKD)	2017	India	Indian	T2DM with DKD	YES	Hospital, Healthy	ADA	Prospective	51.3	50.1
Kavitha, L.	2017	India	Mixed	T2DM	YES	Hospital, Healthy	Doctor's diagnosis	Retrospective		
Ghavimi, R.	2016	Iran	Middle Eastern	T2DM	YES	Healthy, Transfusion Organization	Doctor's diagnosis	Retrospective	51.3	50.2
Eze, I. C.	2016	Switzerland	Caucasian	DM	YES	Healthy	ADA	Cross‐sectional		
Buraczynska, M. (T2DM w/t CVD)	2016	Poland	Caucasian	T2DM without CVD	NA	Volunteers, Healthy	ADA	Retrospective	54.3	
Buraczynska, M. (T2DM w/o CVD)	2016	Poland	Caucasian	T2DM with CVD	NA	Volunteers, Healthy	ADA	Retrospective	65.8	
Saxena, M.	2014	India	Mixed	T2DM	YES	Healthy, Stuff Members	Doctor's diagnosis	Retrospective	49.2	47.8
Karadeniz, M. (T2DM w/t DKD)	2014	Turkey	Middle Eastern	T2DM without DKD	NA	Healthy	Doctor's diagnosis	Retrospective	52.2	54.2
Karadeniz, M. (T2DM w/o DKD)	2014	Turkey	Middle Eastern	T2DM with DKD	NA	Healthy	Doctor's diagnosis	Retrospective	58.3	54.2
Zhang, X.	2011	China	Chinese	T2DM	YES	Healthy	WHO 1999 criteria	Retrospective	57.4	56.8
Bouhaha, R.	2010	Tunisia	Middle Eastern	T2DM	NO	Healthy	ADA	Retrospective	60.6	43.8
Xiao, L. M.	2009	China	Chinese	T2DM	YES	Healthy, Community	WHO 1999 criteria	Retrospective	59.7	51.6
Danielsson, P.	2005	Sweden	Caucasian	T2DM	YES	Healthy	NA	Retrospective	74.0	75.0
Tsiavou, A.	2004	Greece	Caucasian	T2DM	NO	Healthy	Doctor's diagnosis	Retrospective	51.0	44.0
Vozarova, B.	2003	Spain	Caucasian	T2DM	NA	NA	NA	Retrospective	58.6	56.7
Vozarova, B.	2003	USA	Caucasian	T2DM	NA	NA	NA	Retrospective	29.2	63.9

Males (%)		BMI (kg/m^2^)		HbA1c (%)		FPG (mg/dL)		PGG (mg/dL)		TG (mmol/L)		TC (mmol/L)		HDL (mmol/L)		LDL (mmol/L)	
Cases	Controls	Cases	Controls	Cases	Controls	Cases	Controls	Cases	Controls	Cases	Controls	Cases	Controls	Cases	Controls	Cases	Controls
0.640	0.571	34.1	29.4							1.4	1.1	4.2	4.9	1.3	1.5	2.3	3.0
0.343	0.571	34.5	29.4							1.8	1.1	3.9	4.9	1.1	1.5	2.0	3.0
0.300	0.530																
																	
0.368	0.456			7.8													
		26.88		7.59		143.06		199.71		2.39		4.88		1.12		2.69	
		26.55		8.52		154.31		215.16		2.22		4.46		0.97		2.40	
0.186	0.194			8.9		126.5	85.3	203									
0.600	0.670	20.8	24.9														
0.600	0.600	25.88	23.16	7.04	5.89			183.64	122.94								
0.600	0.600	24.3	23.16	9.8	5.89			212.76	122.94								
																	
0.425	0.442	28.5	27.3														
																	
0.460		28.1		7.7						2.10		4.70		1.5			
0.509		26.8		7.9						1.70		4.90		1.2			
0.600	0.662	24.05	23.33			173.81	83.87	272.75	139.65	1.27	1.42	5.74	4.77	1.17	1.25	3.96	1.66
				6.68	5.13	140.53	85.77	193.9	121.3	1.89	1.53	5.36	4.45	1.24	1.37	3.51	2.81
				8.02	5.13	154.77	85.77	218.5	121.3	2.27	1.53	5.35	4.45	1.22	1.37	3.39	2.81
0.576	0.571	24.5	22.4	8.74.7		153	82.8	244.8	102.6	2.69	1.59	4.69	4.86	1.04	1.16	2.51	2.48
0.367	0.715	29.29	27.43			174.42	96.66	167.76		1.73		4.81		1.25			
0.273	0.311	24.22	22.76														
1.000	0.600			6.75	4.65					1.22	0.95			1.2	1.46	2.9	3.35
0.250	0.615																
0.412	0.449																
0.315	0.476																

**Abbreviations:** BMI, body mass index; CVD, cardiovascular disease; DKD, diabetic kidney disease; FPG, fasting plasma glucose; HbA1c, haemoglobin A1c; HDL, high‐density lipoprotein cholesterol; LDL, low‐density lipoprotein cholesterol. Vacant panes denote the unavailability of data; PPG, postprandial glucose; T2DM, type 2 diabetes mellitus; TC, total cholesterol; TG, triglyceride; w/o, without; w/t, with.

### 3.3 Overall analyses: −174G/C polymorphism and T2DM

**FIGURE 2:**
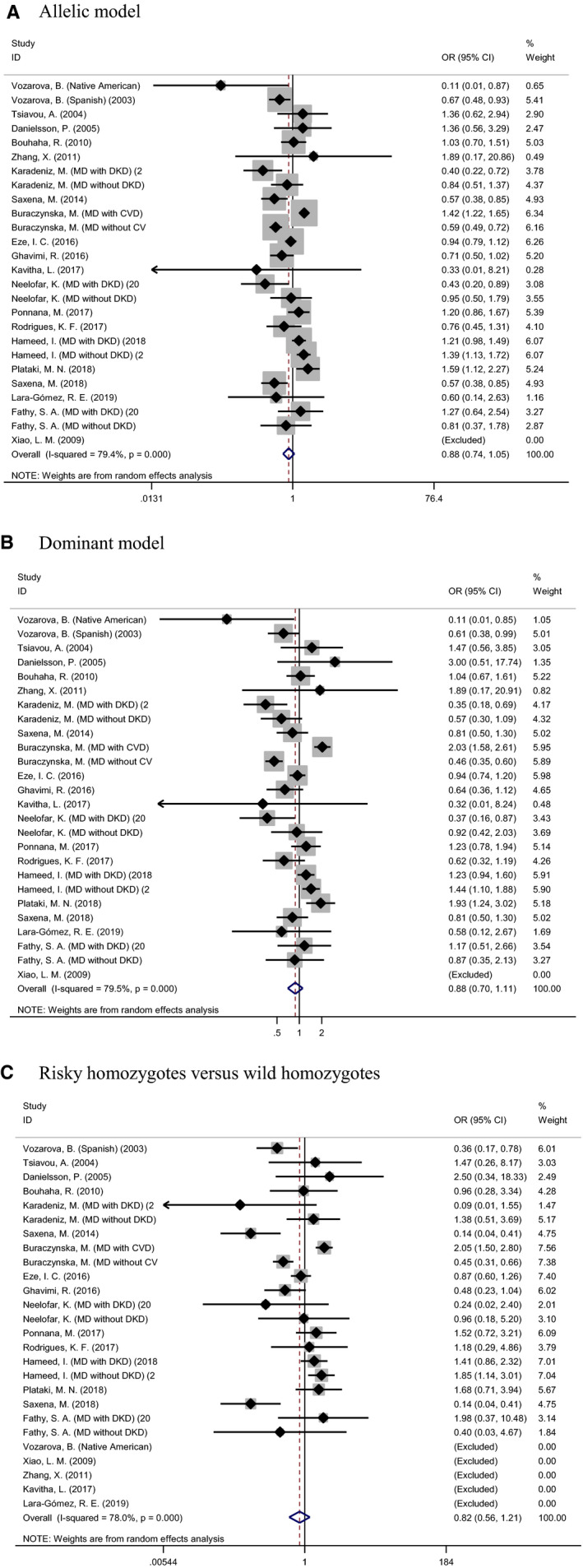
Forest plots of *interleukin 6* gene −174G/C polymorphism associated with type 2 diabetes mellitus under three genetic models

The overall association of *IL‐6* gene −174G/C polymorphism with T2DM was assessed under three different genetic models, as illustrated in Figure 2. The mutation of this polymorphism was related to a reduced risk of T2DM, albeit no detectable statistical significance. For example, carriers of −174CC genotype had an 18% lower risk than those with −174GG genotype (OR: 0.82, 95% CI: 0.56 to 1.21). There was statistically significant between‐study heterogeneity across three genetic models, with the *I* ^2^ around 80% (*P* <.001).

### 3.4 Subsidiary analyses: −174G/C polymorphism and T2DM

Due to significant heterogeneity in overall analyses, a wide panel of subsidiary analyses was carried out separately according to sample sizes, races, countries, diagnostic criteria of T2DM, disease status of patients with T2DM, matched statue and study designs (Table 2). Only subgroups involving at least 2 studies are listed in this meta‐analysis. Heterogeneity was improved in some subgroups, such as in the subgroups with total samples <324 (*I* ^2^: 19.4%), and studies with matched patients and controls (*I* ^2^: 42.0%). In populations with mixed races, the mutation of *IL‐6* gene −174G/C polymorphism was associated with a 37% reduced risk of T2DM (OR: 0.63, 95% CI: 0.46 to 0.86, P: 0.004). No significance was noted for the other subgroups (*P* >.05).

**TABLE 2:** Subsidiary analysis of *IL‐6* gene −174G/C polymorphism in association with T2DM under the allelic model

Subgroups	Number of Studies	OR	95% CI	P	*I* ^2^ (P)
Sample size					
Total sample size <324	12	0.81	0.63 to 1.04	.104	19.4% (.259)
Total sample size ≥324	14	0.91	0.74 to 1.13	.830	87.0% (<.001)
Race					
Caucasian	10	0.98	0.73 to 1.32	.896	86.7% (<.001)
Chinese	2	1.89	0.17 to 20.86	.604	NA
Indian	6	0.96	0.71 to 1.30	.797	77.7% (<.001)
Middle Eastern	4	0.73	0.52 to 1.04	.080	58.0% (.067)
Mixed	4	0.63	0.46 to 0.86	.004	0.0% (.823)
Country					
Asia	15	0.83	0.65 to 1.06	.129	73.0% (<.001)
Europe	7	1.02	0.73 to 1.43	.905	90.4% (<.001)
North America	2	0.30	0.06 to 1.61	.160	44.9% (.178)
Diagnosis of T2DM					
ADA	8	0.93	0.68 to 1.26	.692	88.7% (<.001)
Doctor diagnosis	12	0.91	0.72 to 1.16	.460	69.0% (<.001)
WHO 1999 criteria	2	1.89	0.17 to 20.86	.604	NA
NA	4	0.66	0.43 to 1.00	.052	49.7% (.113)
Disease status in cases					
T2DM	15	0.86	0.67 to 1.09	.217	62.8% (.001)
T2DM with DKD	4	0.74	0.39 to 1.40	.350	83.2% (<.001)
T2DM without DKD	4	1.08	0.78 to 1.48	.650	43.8% (.148)
Matched status					
YES	11	0.83	0.68 to 1.02	.079	42.0% (.078)
NO	2	1.09	0.77 to 1.54	.642	0.0% (.530)
NA	13	0.88	0.67 to 1.15	.342	87.4% (<.001)
Study design					
Prospective	6	0.96	0.71 to 1.30	.797	77.7% (<.001)
Retrospective	20	0.85	0.69 to 1.06	.154	79.4% (<.001)

Abbreviations: 95% CI, 95% confidence interval; ADA, American Diabetes Association; DKD, diabetic kidney disease; *I* ^2^, inconsistence index; NA, not available; OR, odds ratio; T2DM, type 2 diabetes mellitus; WHO, World Health Organization.

### 3.5 Cumulative and influential analyses: −174G/C polymorphism and T2DM

In cumulative analyses, there was no suggestion of significant influence from the first publication on subsequent publications for *IL‐6* gene −174G/C polymorphism associated with T2DM under three genetic models (Figure S1). The influential analyses indicated no significant influence of any one studies on overall estimates under three genetic models (Figure S2).

### 3.6 Publication bias: −174G/C polymorphism and T2DM

**FIGURE 3:**
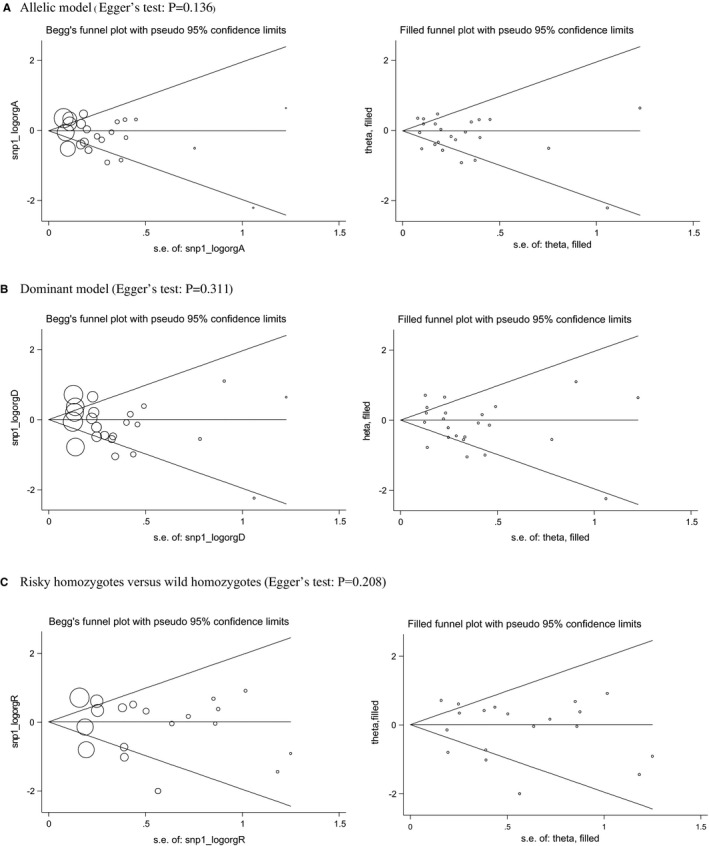
Begg's (the left) and filled (the right) funnel plots of *interleukin 6* gene −174G/C polymorphism associated with type 2 diabetes mellitus under three genetic models

Begg's plots and filled funnel plots are presented in Figure 3 for the association between *IL‐6* gene −174G/C polymorphism and T2DM under three genetic models. The Begg's funnel plots seemed symmetrical, and there was no statistical evidence of publication bias. In addition, there were no theoretically missing studies in filled funnel plots.

### 3.7 Circulating IL‐6 concentrations across −174G/C genotypes

**FIGURE 4:**
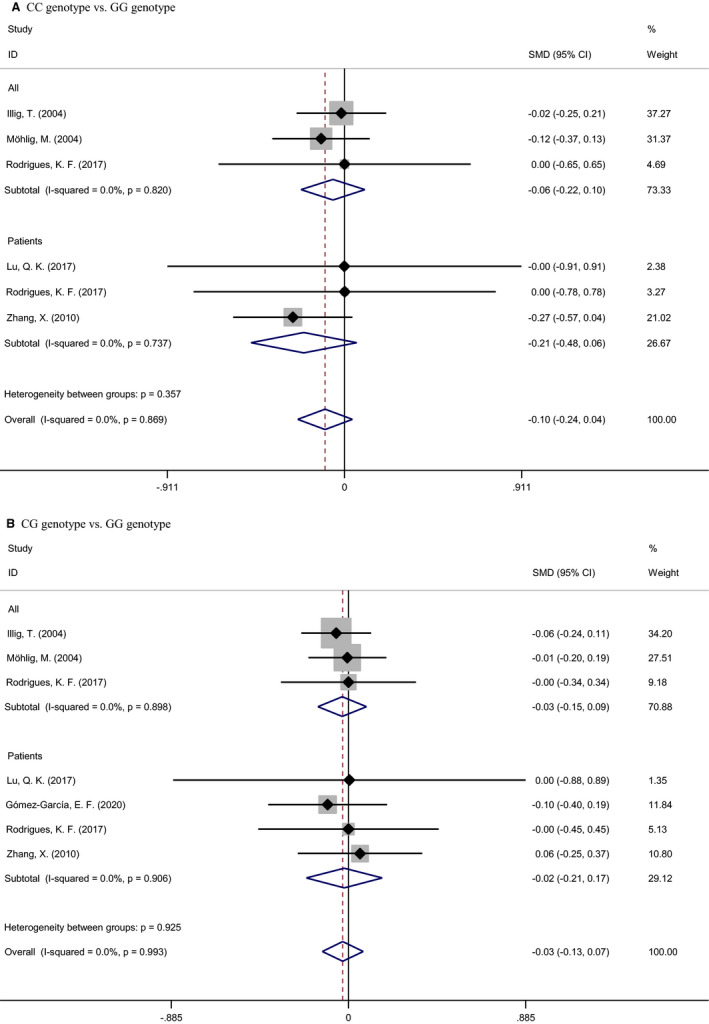
Changes of circulating interleukin 6 concentrations across of the genotypes of −174G/C polymorphism. Abbreviations: SMD, standard mean difference; 95% CI, 95% confidence interval

Figure 4 illustrates the changes of circulating IL‐6 concentrations across the genotypes of *IL‐6* gene −174G/C polymorphism. Taking the carriers of −174GG genotype as a reference group, carriers of −174CC and −174CG genotypes had 0.10 and 0.03 lower concentrations of circulating IL‐6 in pg/mL, albeit no detectable significance.

### 3.8 Other circulating biomarkers across −174G/C genotypes

The changes in other circulating biomarkers, including LDL, HDL, TC, TG, HbA1c, and FPG, across the genotypes of *IL‐6* gene −174G/C polymorphism are separately summarized in Figure S3.

## 4 DISCUSSION

This study was designed to meta‐analytically examine the association of *IL‐6* gene −174G/C polymorphism with T2DM, and circulating IL‐6 changes across −174G/C genotypes. Albeit no detectable significance between this polymorphism and T2DM, our genotype‐phenotype analyses provided suggestive evidence on a dose‐dependent relation between the number of −174G/C mutant alleles and circulating IL‐6 concentrations, indicating possible implication of *IL‐6* gene in the pathogenesis of T2DM. Additionally, our subsidiary analyses revealed that ethnicity and matched status were underlying sources for the obvious between‐study heterogeneity.

In 2006, Qi and colleagues meta‐analysed the association of *IL‐6* gene −174G/C polymorphism with T2DM by pooling the results of 10 articles, and they found that the −174GG homozygotes were not significantly associated with the risk of T2DM compared with −174CC genotype or −174GG plus −174GC genotypes,^45^ in line with the overall findings of the current study. With the accumulating data afterwards, on the basis of the meta‐analysis by Qi and colleagues,^45^ we synthesized the results from 20 eligible articles to examine the association between this polymorphism and T2DM under three genetic models in the current meta‐analysis. Importantly, such a relative large number of eligible studies permitted us to seek underlying sources of heterogeneity. In spite of no detectable significance in both overall and subsidiary analyses, we observed that the association between *IL‐6* gene −174G/C polymorphism and T2DM was more obvious under the dominant model and the relation between circulating IL‐6 concentrations across −174G/C genotypes followed a dose‐dependent manner. We cannot preclude the possibility that *IL‐6* gene −174G/C polymorphism may not, by itself, exhibit significant predisposition to T2DM, mainly because its effect is small and may be dependent on the presence of other mutations. We agree that further large, well‐designed, prospective investigations are warranted to confirm the susceptible role of *IL‐6* gene in the pathogenesis of T2DM.

Extending the findings of previous meta‐analysis by Qi and colleagues,^45^ we noticed that race and matched status were underlying causes of previously conflicting reports. Indeed, there is a wide recognition that the development of T2DM is complex, and divergent genetic determinants or linkage profiles might account for these differences.^46^, ^47^ A variant may be a candidate locus for T2DM in one ethnic group, but not in another, which was further reinforced in the current meta‐analysis, when analysing the association of *IL‐6* gene −174G/C polymorphism with the risk for T2DM upon stratification by races. Another important aspect is the confounding that results from unmatched cases and controls. In fact, our effect‐size estimates in the current meta‐analysis were derived from allele or genotype counts, overlooking the consideration of other confounding factors, such as age, gender and lifestyle factors.^48^ The disparities in the findings of previous studies may be attributable to unaccounted residual confounding.^49^ A potentially power approach to avoiding residual confounding is through Mendelian randomization.^50^ Due to the non‐significant observations in genotype‐disease and genotype‐phenotype analyses, Mendelian randomization cannot be further conducted, as this approach requires genotypes that influence the variable of interest are directly related to the outcome.

The contribution of IL‐6, as a pro‐inflammatory cytokine to the pathogenesis of T2DM, is biologically plausible.^51^ Actually, IL‐6 acts via two distinct signalling pathways in the development of diabetes, that is, classic signalling and trans‐signalling. The final biological effects of these two signalling modes that lead to activation of the same receptor subunit are completely different.^23^ Knockout experiments showed that the expression of IL‐6 was significantly elevated in insulin‐resistant individuals.^52^ Although IL‐6 is an indicator of inflammation, the study by Mauer and colleagues demonstrated that it can limit inflammation by promoting the alternative activation of macrophages to curb inflammation.^53^ In addition, IL‐6 is considered to be involved in the development of inflammation, insulin resistance, as well as β‐cell dysfunction.^23^ The interaction between IL‐6 and TNF‐α can exacerbate oxidative stress and reduce phosphorylation of endothelial nitric oxide synthase (eNOS), which may cause various complications.^54^ On the basis of above evidence, it is reasonable to speculate that *IL‐6* gene is a possible candidate in susceptibility to the development of diabetes.

Several limitations should be acknowledged for the current meta‐analysis. The first limitation lied in the analysis of only one polymorphism in *IL‐6* gene. The second limitation was that only retrieved articles in English were analysed in this study, and the ‘grey’ literature was not included. The exclusion of ‘grey’ literature from meta‐analysis may result in an overestimate of an association impact by an average of 12%.^55^ The third limitation was about publication bias. Although there was a low probability, the possibility of missing small or negative studies that had not yet been published was still existed. The fourth limitation was about heterogeneity. Although a set of auxiliary analyses had been conducted, the heterogeneity was still significant in some subgroups, which limited the interpretation of combined risk estimates.

Taken together, albeit no detectable significance between *IL‐6* gene −174G/C polymorphism and T2DM, our genotype‐phenotype analyses provided suggestive evidence on a dose‐dependent relation between the number of −174G/C mutant alleles and circulating IL‐6 concentrations, indicating possible implication of *IL‐6* gene in the pathogenesis of T2DM. For practical reasons, our hope is that this meta‐analysis will not represent just another endpoint of investigations, instead of a start to clarify the association of other genetic defects in *IL‐6* gene with the risk for T2DM, as well as to elucidate the underlying molecular mechanisms of circulating IL‐6 concentrations in the onset and progression of T2DM.

## CONFLICT OF INTEREST

The authors declare that they have no conflicts of interest.

## AUTHOR CONTRIBUTION

**Hao Cheng:** Data curation (equal); Formal analysis (equal); Writing‐original draft (lead). **Wenbin Zhu:** Data curation (lead); Writing‐original draft (equal). **Mou Zhu:** Methodology (lead). **Yan Sun:** Methodology (equal); Project administration (equal). **Xiaojie Sun:** Methodology (equal); Project administration (equal). **Di Jia:** Project administration (lead). **Chao Yang:** Data curation (equal); Project administration (equal). **Haitao Yu:** Supervision (equal); Writing‐original draft (equal); Writing‐review & editing (equal). **Chunjing Zhang:** Conceptualization (lead); Supervision (lead); Writing‐original draft (equal); Writing‐review & editing (equal).

## Supporting information

Supporting information

## Data Availability

Data involved in this study are available upon reasonable request.
